# What are the views of those participating in a trial investigating acute post-traumatic benign paroxysmal positional vertigo? A qualitative study

**DOI:** 10.1080/02699052.2024.2435952

**Published:** 2024-12-03

**Authors:** Rebecca M Smith, Bithi Sahu, Caroline Burgess, Jenna Beattie, Abby Newdick, Vassilios Tahtis, Jonathan Marsden, Barry M Seemungal

**Affiliations:** a Centre of Vestibular Neurology, Imperial College London, London, UK; b Population Health Sciences, Kings College London, London, UK; c Occupational Therapy, Imperial Healthcare NHS Foundation Trust, London, UK; d Physiotherapy, St George’s Hospital NHS Foundation Trust, London, UK; e Occupational Therapy, Kings’ College Healthcare NHS Trust London, UK; f School of Health Professions, University of Plymouth Plymouth, UK

**Keywords:** Benign paroxysmal positional vertigo, traumatic brain injury, qualitative, feasibility trial, rehabilitation

## Abstract

**Objective:**

The purpose of this study was to explore the experiences of patients and healthcare professionals participating in a randomized feasibility trial exploring the management of acute post-traumatic benign paroxysmal positional vertigo (BPPV), and to establish the acceptability and tolerability of BPPV assessment and treatment procedures.

**Methods:**

This was a multi-center qualitative study nested within a wider randomized feasibility trial. Purposive sampling was used to gather a cohort of healthcare professionals and patients from three major trauma centres in London, UK. Data were gathered using semi-structured interviews and were analyzed using Framework analysis.

**Results:**

Fifteen healthcare professionals and 26 patients participated. Patients and healthcare professionals reported acute BPPV diagnosis was acceptable and practicable. However, divergence was noted regarding views of randomization. Participants proposed several research delivery and protocol modifications for a future trial, including changes to study design and outcome measures.

**Conclusions:**

Healthcare professionals and patients participating in a multi-center qualitative study felt post-traumatic BPPV was feasible and acceptable to diagnose and treat acutely. Findings from this study will enhance the content and delivery of a future trial and may assist in influencing the development of clinical practice guidelines.

## Introduction

Among the physical, psychological and socioeconomic impairments caused by traumatic brain injury (TBI), vestibular dysfunction is common (1,2). Vestibular dysfunction (attributed to damage to the peripheral [inner ear] or central [brain] vestibular system) manifests in symptoms of dizziness or imbalance, which can persist (3,4), and are associated with reduced health-related quality of life (5,6) and delayed return to work (7). The most frequent cause of acute post-traumatic peripheral vestibular dysfunction is Benign Paroxysmal Positional Vertigo (BPPV) (1). Critically, previous research notes that BPPV is associated with falls (8), which due to the associated mortality risk in TBI survivors (9), provides a rationale for timely management. Despite this, post-traumatic BPPV is not routinely managed acutely by ward healthcare professionals (10).

Delays to managing BPPV are problematic, resulting in a reduced quality of life (11) and a higher burden of symptoms, even following treatment (12). Barriers to routine management of post-traumatic BPPV included role- and knowledge-based factors, alongside concerns regarding the practicability and tolerability of assessment and treatment procedures (10). To further compound these challenges, current national clinical guidelines for early management of head injury do not advocate routine BPPV assessment or treatment (13), due to a paucity of evidence relating to the effectiveness of interventions. Indeed, there are currently no acute, prospective randomized trials investigating the effectiveness of treating post-traumatic BPPV.

Research exploring acute assessment and treatment of post-traumatic BPPV is therefore necessary. Feasibility studies are an important pre-cursor to more definitive effectiveness trials and allow researchers to explore uncertainties in a less costly format (14). Uncertainties pertaining to a trial exploring acute management of post-traumatic BPPV include (i) the acceptability of trial procedures including randomization, (ii) the appropriateness of existing outcome measures and (iii) the practicability and acceptability of BPPV procedures. The inclusion of qualitative research at the feasibility stage is critical due to the benefits it may afford a future trial (15), including heightening external validity through use of appropriate measures, increasing cost-effectiveness by only selecting optimized interventions and aiding interpretation of trial findings (16).

The objective of this study was to explore the experiences of patients and healthcare professionals participating in a feasibility trial exploring the management of acute post-traumatic BPPV, and the acceptability of assessment and treatment procedures.

## Materials and methods

This was a multi-center qualitative study nested within a wider randomized feasibility study. A brief description of the feasibility study design follows. Hospitalized adults with traumatic brain injury (defined using the Mayo classification (17)) were eligible to participate. Patients were excluded if they had orthopedic or vascular instability precluding BPPV testing, were medically unstable, had a current or a history of substance misuse, pregnancy, a prescription of Phenytoin or a Glasgow Coma Score of <14 at the time of assessment. Regardless of complaints of vertigo, all consenting patients underwent testing for posterior and horizontal canal BPPV via Dix Hallpike and Supine head roll tests. Patients were tested on average 6 days following their injury. BPPV was diagnosed using Bárány Society criteria (18). Consented patients testing positive for BPPV were randomized to one of the three interventions (re-positioning maneuvers, Brandt-Daroff exercises and advice). Given the lack of evidence relating to the effectiveness of maneuvers in post-traumatic BPPV and the uncertainty surrounding the practicalities of completing maneuvers, this study employed therapist-led maneuvers, patient-led Brandt Daroff exercises and advice (which constituted usual care in many major trauma centers). Briefly, re-positioning maneuvers were delivered by therapists and followed clinical practice guidelines (19). More specifically, Epley and Semont maneuvers were used for posterior canal BPPV and the log-roll for horizontal canal BPPV. Those allocated to the Brandt Daroff group received two therapist supervised sessions in the hospital and were also instructed to continue with the exercises twice daily for 2 weeks. Patients in the advice group were provided with two in-patient sessions of verbal and written advice on standing and moving safely. Fuller details on the interventions can be found in the published protocol (20) and trial results (21). Following trial completion, patients and healthcare professionals were interviewed to explore their experiences and the acceptability of acute BPPV management.

## Study design

A qualitative approach was deemed useful to explore the experiences of healthcare professionals and patients during a feasibility study. The Framework approach was used to gather and analyze data. This methodology was selected given its use in similar studies exploring the experiences of clinical trial participants (22–24).

## Setting and participants

This study was conducted across three major trauma centres in London, UK. Participants were healthcare professionals and patients taking part in the randomized feasibility trial. Healthcare professionals included physiotherapists, occupational therapists, and research nurses. Therapists (inclusive of physiotherapists and occupational therapists) had a variety of trial roles including recruitment, randomization, BPPV assessment, and treatment and outcome measure completion. The rationale for therapist-led assessment and treatment follows previous qualitative work (10). The protocol for therapist training has previously been published (21,25). Research nurses completed study procedures pertaining to recruitment and outcome measure completion. Purposive sampling was used to gather a sample of patients across all three treatment arms and all sites. Patients were invited by the chief investigator to participate at their final trial follow-up appointment. Two patients who withdrew from the study also provided reasons for withdrawal. Such data were not formally analyzed but are included in the findings.

Purposive sampling was used to obtain a sample of healthcare professionals (inclusive of physiotherapists, occupational therapists, and research nurses) across all trial sites and with a range of trial roles (i.e., screening, consent, assessment, treatment, randomization, and outcome measure completion). Healthcare professionals were invited by e-mail to participate. Guidance from previous studies using Framework analysis (26,27) and discussions with the research team were used to determine appropriate sample sizes.

## Data collection

Individual, semi-structured interviews, using a topic guide, were conducted by two research team members (RMS and BS). Interviews were conducted face-to-face in the workplace or on Microsoft Teams according to participants’ preference. Interviews were audio-recorded and transcribed verbatim. All patient interviews were conducted by the same researcher (RMS), and all healthcare professional interviews were conducted by the same researcher (BS).

The theoretical domains framework (TDF) was used to inform separate topic guides for healthcare professional (Supplementary material 1) and patient (Supplementary material 2) interviews. Theoretical frameworks, such as the TDF, are used to identify and enable areas of behavior change and develop implementation interventions (28,29). Here, the TDF was used to identify and explore (i) barriers or facilitators to the trial, (ii) implementation of the intervention and (iii) potential modifications to trial delivery.

## Data analysis

Data were analyzed inductively using the Framework approach; a series of five steps whereby codes are applied to each transcript and reduced into groups or themes (30). Separate frameworks were utilized for healthcare professional and patient data due to the different nature of the populations. To heighten the rigor and transparency of the results, two research team members coded the transcripts. Framework drafts were discussed among the research team and refined until a consensus was reached. An example of a charted theme is provided in the supplementary material (Supplementary material 3).

## Ethics

Ethical approval for this study was obtained from the East of England Research Ethics Committee (19/EE/0052).

## Results

Results are reported in accordance with the Consolidated reporting guidelines for Qualitative research (COREQ) (31). Quotes are provided to illustrate themes and are followed by the pseudonym, profession or treatment group, and trial site of the participant.

## Views and experiences of healthcare professionals

Twenty healthcare professionals were invited to participate. Five declined or did not reply to the e-mail. Fifteen healthcare professionals completed interviews (Table 1). On average, healthcare professionals had 5 years’ experience in trauma and were a senior therapist or research nurse. Interviews lasted on average 37 minutes.

**Table 1.** Demographics of healthcare professionals participating in the study.

Healthcare professional (n)	Number interviewed (n)	Age (mean ± SD)	Females (n, %)
Physiotherapist (7)			
Site A	3	33 ± 4	2
Site B	3	30 ± 3.5	3
Site C	1	27	1
Occupational therapist (4)			
Site A	1	37	1
Site B	0	N/A	0
Site C	3	39 ± 4.6	2
Research nurse (4)			
Site A	4	33 ± 5.25	3

**Figure 1.**
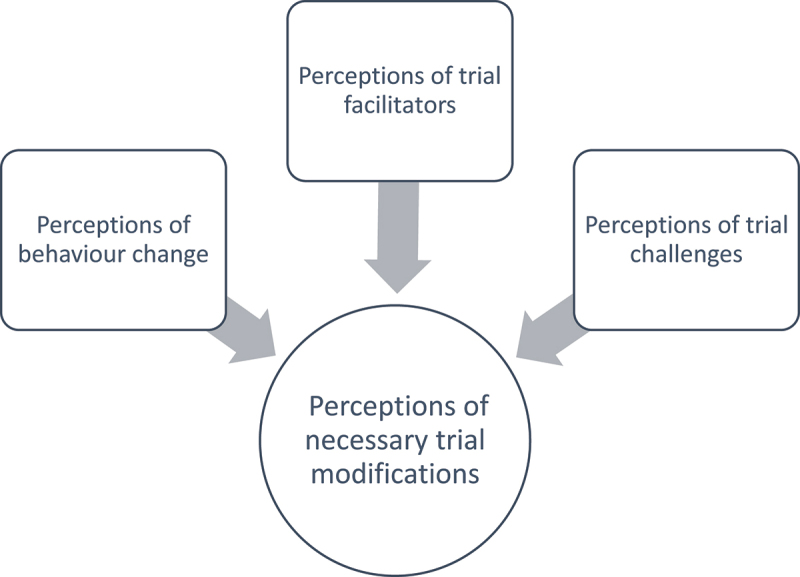
Main themes relating to healthcare professionals’ experiences of trial participation.

Four key themes relating to healthcare professionals’ experiences were noted: Perceptions of behavior change, Perceptions of trial facilitators, Perceptions of trial challenges, and Perceptions of necessary trial modifications. The three former themes were felt to feed into necessary modifications for a future trial (Figure 1).

## Perceptions of behavior change

For many therapists, assessment and treatment of BPPV was a new skill requiring role extension and clinical behavior change. Following training, however, therapists felt they had sufficient knowledge, skills, and confidence to manage BPPV. Notably, BPPV management was perceived to be an appropriate skill for therapists’ role, although for occupational therapists, this had not been the case historically. Specific training and knowledge had enabled them to some extent, to dispel stigma around undertaking these new skills. Training both physiotherapists and occupational therapists was felt to result in service-level benefits. Brain-injured patients who presented with minor physical impairments could therefore be assessed solely and holistically by an occupational therapist (including assessment for BPPV).

‘There’s a bit of a stigma around OTs assessing dizziness as well and it was always, sort of, the physios doing the dizziness assessments… I think the study sort of enabled them to understand that it doesn’t have to just be a physio, that it can be OTs as well.’ [KC0408, Occupational Therapist Site C]

Therapists felt improvements in their knowledge, skills, and confidence resulted in (i) an enhanced ability to manage more complex BPPV cases, (ii) improved awareness of appropriate medication use for BPPV-induced vomiting, (iii) more effective communication with patients about BPPV and (iv) knowing the limitations of their practice.

Reported advantages to clinical teams included heightened awareness of the need to manage BPPV the ability of therapists to utilize these skills, and the translation of such skills to different clinical areas. At one site, participants reported therapist-led BPPV management reduced referrals to visiting specialists.

‘We reduced the amount of referrals needed to our neuro-otology service. I think we also reduced the length of stay. We haven’t proven that, but I think we probably have because of early assessments and things. I think we have also massively increased awareness amongst the medical team to be wary of vestibular dysfunction…They are not just sending them home.’ [SM2506, Physiotherapist, Site A]

Therapists perceived managing BPPV had a positive impact on patients, noting that objective screening resulted in more BPPV diagnoses (as patients without symptoms, or a vestibular agnosia, were picked up). Therapists also witnessed treatment-related improvements in patients’ symptoms and function, which facilitated discharge. Interestingly, one participant reported some uncertainties regarding the optimal timing of treatment.

Therapists felt it was feasible to assess and treat post-traumatic BPPV acutely. Due to pain, headaches, or nausea, waiting as long as possible to assess patients, whilst they were still in hospital, was felt to be appropriate. Generally, acute diagnosis and treatment was perceived to (i) enable other teams to complete their assessments in a timely manner, (ii) provide patients with a diagnosis and validate their symptoms, (iii) avoid long delays to accurate diagnosis and treatment, and (iv) ensure risk factors for falls, such as BPPV, were addressed prior to discharge. When considering implementation of routine assessment and treatment into clinical practice, therapists were positive about the prospect, but noted further data would be required, including endorsement from local or national clinical guidelines.

## Perceptions of trial facilitators

Across all sites, therapists reported positive aspects of being involved in the trial. They enjoyed having ownership of the research, with early involvement in protocol design perceived to be rewarding and helpful. A range of resources were cited to facilitate their involvement. For example, at one site, therapists worked in a split research/clinical role which was perceived to be hugely advantageous as it afforded flexibility and created a team research ethos. The other two sites did not have split research/clinical posts but did have funded time to complete screening, consent, and outcome measures, activities which were perceived to be the most time-consuming. Therapists solely completing trial-related BPPV assessments and treatments, in conjunction with their clinical caseload, felt this was manageable.

‘I think having the support of the research nurses for consenting and doing some of the other assessments, some of the outcomes measures was really helpful because I think that was one of the more time-consuming bits.’ [SM0403, Physiotherapist, Site A]

Having multiple staff members trained to assess and treat patients was perceived to be helpful, to increase capacity, and cover staffing shortages. Written information covering key assessment and treatment points was noted to be useful. Other key resources included access to experts for clinical queries, supportive managers, networking, and peer support from other sites.

## Perceptions of trial challenges

Therapists and research nurses reported general and study-specific challenges. General challenges comprised time pressures and the capacity of staff to complete trial processes. Time pressures were particularly noticeable when completing trial procedures for patients with mild brain injury and short admissions. Further, time and capacity were cited as barriers for therapists completing re-assessments or second treatment sessions.

Study specific challenges were mainly around recruitment, outcome measures, and randomization. Participants across all sites felt patients’ cognition and capacity were significant barriers to consent due to confusion, fatigue, or difficulties with attention. Interestingly, research nurses felt patients with BPPV were particularly difficult to consent, as they felt nauseous, dizzy, or struggled to read trial information.

‘Patients that have the condition, they really struggle to pay attention to you … I feel the patients that have BPPV are less likely to consent to go into the trial and that’s a huge barrier because then you’re not getting a really representative population’ [RN2602, Research nurse, Site A]

Additionally, the unexpected nature of trauma was thought to result in patients not being primed for being asked to participate in research, and thus the process could be somewhat overwhelming.

Across all sites, the number and nature of some of the outcome measures were felt to be a challenge. Indeed, participants cited that the subjective dizziness questionnaires (particularly the dizziness handicap inventory) were too community-focused, leading to patients not answering questions accurately or not at all. The concept of randomization also presented challenges. Where therapists were already accustomed to treating BPPV with re-positioning maneuvers, there was an understandable reluctance to change practice, notwithstanding the lack of evidence. Despite a general understanding of the need for randomization, some therapists felt particularly uneasy about delivering the advice arm. This was due to feeling somewhat responsible that patients may return home feeling dizzy and at risk of falls. However, there was an overarching sense that the study was safe and had appropriate safety nets.

‘Someone that’s a high fall risk you might feel a little bit uneasy giving them advice to go home with when you might feel like that could put them at risk of falling. But you have to remind yourself, that this is for a study because actually we don’t have a strict evidence base’ [SG2502, Physiotherapist, Site B]

Challenges were noted in aspects of assessment and treatment. Pain, braces, collars, or casts were felt to pose barriers, as well as lack of light in cubicles to confidently interpret nystagmus. Nausea and tolerability of treatment appeared to vary and create difficulties for some patients, although therapists reflected that patients were grateful when treatments were successful. Therapists noted that Brandt-Daroff exercises relied on patient compliance and motivation, as well as needing a certain level of cognition, flexibility, and balance.

## Perceptions of necessary trial modifications

Participants across all sites felt positive about a future trial. This was due to (i) a perception that acute management of BPPV was feasible, (ii) there had been a positive impact of managing BPPV acutely, and (iii) a future trial would provide further data and facilitate BPPV management becoming routine practice.

‘I think we need it, and I welcome it, and I think it would be a very important study. I think it could have profound impact on how we deliver acute trauma care nationally, so I think we should run it’ [KC0104, Occupational Therapist, Site C]

Therapists made several recommendations for a future trial including building in funding for those working on research activities and a study-specific research therapist, located and embedded on the trauma ward, to undertake research activities. This could reduce time inefficiencies and improve communication. Notably, participants felt it would be important to train a separate, larger group of staff to complete assessment and treatment procedures to increase capacity and in the knowledge it would be a skill used in routine practice after cessation of the study.

Participants felt improvements could be made to recruitment processes, including widening the inclusion criteria, using leaflets to prime patients for research, using alternative methods of providing patient information, and consenting only those testing positive for BPPV.

‘If there was a nominated consultee consent but only for maybe the test and outcome measures to be done, and any follow up you would need patient consent.’ [RN2602, Research Nurse, Site A]

There was a general feeling that a future trial would benefit from fewer and more relevant outcome measures. There was also consensus that continued or rolling training throughout the trial would facilitate (i) training of rotational staff and thus mitigating loss of capacity during the study period, (ii) maintenance of competency and confidence levels, and (iii) preservation of interest and motivation. Suggested strategies included educational forums, videos of eye movements, and joint sessions or access to experts for clinical queries.

## Views and experiences of patients

Forty patients were invited to participate. Of those, 14 declined to take part. Twenty-six patients consented and completed interviews (Table 2). The majority of patients (69%) had suffered a moderate-severe TBI according to the Mayo classification (17). Patients had an average score of 26.2 ± 2.9 on the Montreal Cognitive Assessment.

**Table 2.** Demographics of patient participants.

Treatment arm (n)	Number (n)	Male (n)	Age (mean, SD)	Resolved BPPV at interview (n)
Manoeuvres (9)				
Site A	5	3	53 ± 17	4
Site B	2	1	35 ± 18	0
Site C	2	2	58 ± 37	2
Brandt-Daroff (8)				
Site A	4	2	68 ± 9	1
Site B	3	2	57 ± 5	2
Site C	1	1	55	0
Advice (9)				
Site A	2	2	74 ± 10	2
Site B	6	6	58 ± 18	4
Site C	1	1	48	0

Data analysis noted three main themes: Timing of trial procedures, content and delivery of trial procedures, and proposed modifications to trial procedures.

## Timing of trial procedures

### Timing of recruitment

There were mixed feelings about being asked to participate in a trial during the acute stage of a TBI. For some, it was a period with many clinical assessments meaning it was somewhat difficult to process trial information. Some patients recounted they needed to read the information back weeks later as they were unable to recall aspects of the trial. Echoing research nurse views, one participant noted there was no prior communication that a researcher might discuss trial participation with them, and therefore being approached for consent was a surprise. However, others felt being asked to participate in a trial during this acute stage was acceptable as they hoped to better understand the cause of their symptoms, which was felt to be facilitatory for recovery.

### Timing of diagnosis

There were strong feelings that diagnosing BPPV acutely was beneficial, both intrinsically and extrinsically. Intrinsic benefits included reassurance and heightened confidence due to knowledge (i) there was a reason for their dizziness, (ii) there was a treatment plan, and (iii) any risky activities encountered on returning home (e.g., stairs, showering, shopping) could be mitigated. Importantly, a diagnosis also led to validation of symptoms and appropriate empathy and support from healthcare professionals. Extrinsic benefits of an inpatient diagnosis were (i) the convenience of being in hospital and not needing an outpatient appointment, (ii) avoiding delays to diagnosis and treatment, and (iii) a benchmark of dizziness severity to enable accurate monitoring and follow-up.

‘If I didn’t know what it was and I left the hospital and I had a really intense dizzy period, it would be a bit more frightening thinking that there might be something else wrong, Whereas, you know understanding what BPPV is, it’s obviously just, it’s the crystals in the ear. Whereas you know I’d probably if I didn’t know it was that I might like catastrophise the situation’ [KC2307, Site C, Advice]

Notably, some participants did caveat an early diagnosis by waiting as long as possible in hospital to recover from nausea, pain, or surgery, prior to completing assessments. Assessments undertaken on the day of discharge were not preferable due to procedures provoking nausea and exacerbating fatigue.

### Timing of treatment

Views on the timing of treatment were mixed and appeared to differ according to injury severity, dizziness severity, or treatment group allocation. Patients in the advice arm felt somewhat conflicted regarding treatment timing. One patient in the advice group was unsure whether they would have tolerated repositioning maneuvers acutely, due to severe nausea and vomiting after treatment at 12-week follow-up. Conversely, they also noted dizziness was impacting their daily activities, and therefore resolution of BPPV would have been helpful slightly sooner than 12-week follow-up. Others in the advice group noted that due to complex spinal or limb injuries, they were somewhat sedentary at home and thus experienced fewer dizzy episodes. Waiting for treatment or not having active treatment acutely did not impact them to the same extent.

‘I had a lot of other injuries and a lot of other things to think about … the fact that I didn’t really have to think about it much was really useful and meant that I wasn’t, you know, putting all my thinking time and worry into something.’ [SM2312, Site A, Advice group]

## Delivery and content of trial procedures

### Trial information and taking part

Views on trial information were somewhat discordant, some reported it facilitated understanding of the protocol and decisions to participate, whilst others were unable to recall aspects including randomization. Patients cited different reasons for participating, either personal (linked to concerns about symptoms) or altruistic factors (wanting to help others). Notably, some reported participation was not dependent on the presence of dizziness. Indeed, a proportion had not expected to have BPPV, as up until the assessment they had been sedentary and asymptomatic.

### Randomization

Generally, patients reported that the principle and process of being randomized to interventions was acceptable, noting that individualized treatments reduced concerns about uniform interventions. Facilitators to positive feelings about randomization were (i) the lack of research regarding ‘optimal’ treatment, (ii) knowledge of what to expect within the trial and (iii) safeguards, including withdrawal (these appeared to be particularly important for the advice group).

‘I think because it was explained to me that if I was part of a treatment group where the treatment didn’t work for me, I would be able to try the other treatments at the end of the study. I was feeling pretty confident that I’d get, you know, treated either way, kind of thing….’ [KC2307, Site C, Advice group]

Patients voicing concerns about randomization were evenly spread throughout the groups. Some linked their discomfort at randomization to their symptom severity and the impact of dizziness on their wellbeing, or the potential impact of ineffective treatment on their overall recovery.

### Assessment and treatment

The diagnostic test for BPPV was generally perceived to be safe and well explained. Patients noted it caused short-lived nausea and dizziness, but not pain. Those in the advice group generally viewed their treatment positively, citing the advice as clear, helpful, and straight-forward. Participants completing the Brandt-Daroff exercises noted they were easy and safe to complete independently. Some noted the exercises induced dizziness, although this was tolerable and transient. Despite varying exercise adherence, there was a reported positive effect of the exercises on symptoms. One patient noted that having a task ‘to do’ in the hospital (i.e., the exercises) was useful and engaging. Respondents in the maneuver group reported treatments felt safe and well explained. Transient eye movements, nausea, and dizziness during the maneuver were described as somewhat uncomfortable, but were outweighed by successful treatment. Patients expressed a preference that maneuvers should not be completed on the day of discharge.

‘It was easy to do, it was safe to do, so it wasn’t a problem to do it…I did complete the course of exercises’ [SM2403, Site A, Brandt-Daroff group]

### Outcome measures

Due to the complexity of their injuries (i.e., involvement of brain, limb, and spine), participants noted it was not always possible to disentangle which symptoms or feelings were related to BPPV. Similarly, respondents were aware their answers were also impacted by external factors, such as family, work, and other general feelings, unrelated to their TBI. To a lesser extent, participants with mild TBI noted that several questionnaires were irrelevant, particularly those regarding physical impairments.

## Proposed modifications to trial procedures

Several recommendations were made for a future trial. Those completing Brandt-Daroff exercises noted adherence and confidence could be improved by (i) more supervised sessions with hospital therapists, (ii) a phone or video call post-discharge to reiterate and reinforce completion of the exercises, and (iii) online links to a video of the exercise and a way of recording adherence, accessible to patients and researchers. Participants also suggested questionnaires might be modified, removed, or made more specific to the severity of the injury. A final recommendation was to modify the delivery and content of information about BPPV and randomization to interventions. Indeed, some noted the verbal information regarding the nature of BPPV, although well explained, was hard to understand because it sounded far-fetched or unbelievable.

‘I found it difficult to understand; crystals in the ears, give me a break, but after a little while, even after a few minutes, I understood, that makes sense. But I can see many people not understanding it.’ [SG0806, Site B, Brandt-Daroff group]

### Study withdrawal

Two patients withdrew from the advice group, one of whom noted the explanation regarding different treatment groups was insufficient, leading to an expectation of more active treatment. Due to having multiple other injuries they preferred to have active treatment sooner rather than later. This view differed from that of other patients in the advice group who were happy to wait for treatment. The other patient who withdrew from the study felt it was preferable to have treatment in hospital to avoid having to return for further appointments.

## Discussion

This multi-center qualitative study explored the experiences of patients and healthcare professionals participating in a trial investigating the management of BPPV in acute TBI. Key findings are noted below, alongside data-driven recommendations for a future trial.

### Participants’ views on the feasibility of managing post-traumatic BPPV

This is the first study to demonstrate that healthcare professionals and patients viewed therapy-led acute post-traumatic BPPV assessment and treatment as both practicable and acceptable. Patients and healthcare professionals also had congruent views on the benefits of acute diagnosis, noting a positive cascade of knowledge, understanding, and confidence. This is in line with other research noting the importance of a diagnosis for patients with vestibular disorders to facilitate recovery and positive health-related beliefs and behaviors (32–34). Thus, alongside previous studies noting the consistency and accuracy of therapist-led BPPV management (35) and the impact on onward referrals to specialists (25), there is reasonable evidence to suggest (i) trauma ward therapists can manage post-traumatic BPPV and (ii) provision of a diagnosis acutely is beneficial for patients. However, as the findings suggest, it would be important to embed these skills into clinical roles more permanently. Guidelines or pathways could be used for this purpose (36), although uptake can be hindered by factors related to implementation, as well as organizational and individual factors (37). Current national early head injury guidelines do not stipulate early vestibular assessment or treatment (13) and thus further clinical trials may be necessary to inform such guidelines. Future work may benefit from engaging with healthcare professionals in the design of implementation strategies to improve uptake (37), whilst the importance of qualitative research to establish patients’ experience in shaping clinical guidelines may also be critical (38,39).

### Participants views on randomization

Divergence was noted between healthcare professionals and patients in their views of randomization, specifically around allocation to the advice group. Healthcare professionals had concerns regarding patients’ safety following discharge, whilst patients in the advice group who completed the study reported little impact on their recovery, with some noting active treatment acutely would not have been possible. Notable, however, were the two withdrawals from the advice group, seemingly related to the lack of active intervention. Patient disappointment at a lack of intervention has been cited in previous studies as a reason for withdrawal (40), whilst other research notes the importance of providing information pertaining to randomization to ensure expectations are managed (41,42). The concerns voiced by healthcare professionals in the present study are not dissimilar to those noted elsewhere, whereby apprehensions regarding treatment preferences, and individual patients were reported barriers to recruitment (43). Authors recommended revisiting trial information and ensuring practical support is provided to participating staff. A future randomized trial of BPPV interventions may need to evaluate the need for alternative designs such as multi-arm, multi-stage trials whereby the removal of a treatment arm or changing the allocation of patients to a trial arm can take place during the trial, depending on ongoing analyses.

### What changes should be made to a future trial?

Healthcare professionals and patients suggested changes to outcome measures. In particular, the subjective dizziness questionnaire (Dizziness Handicap inventory [DHI]) was considered inappropriate due to its community-based nature. Indeed, recent research noted that despite its widespread use, there were insufficient data regarding content validity and reliability (44). Further, a review of a range of vestibular outcome measures, including the DHI, concluded no measures were appropriate for use in acute settings, such as the Emergency Department, due to limited psychometric properties (45). These findings suggest the need for a new acute outcome measure, rigorously designed to evaluate the impact of acute dizziness (such as BPPV) on patients’ function and activities of daily living.

Recruitment processes were also an area highlighted for improvement. Previous research evaluating recruitment strategies noted the timing of approach in relation to the injury or diagnosis may be key, whilst older adults may be less likely to participate due to feelings of mistrust (46). Timing may well have been relevant in this study as some patients reported being overwhelmed by information in the acute stage of their injury. Interestingly, research notes that older adults have the highest incidence of TBI (47) and may be under-represented in TBI studies (48), due to factors including more circuitous diagnostic routes (49). Future trials may wish to consider recruitment outside of major trauma wards to ensure a greater representation of older adults, as well as strategies including the provision of extra time, training on communication for researchers, and appropriately designed patient information (48,50).

Recently, written patient information sheets have been replaced with digital or multimedia versions (51,52), which although did not directly increase recruitment, were felt to be easier to understand (52). This would be important for a future BPPV trial given therapists reported BPPV was hard to explain, and patients reported information around BPPV was not easily understood. Patients and healthcare professionals expressed views that modifications to the delivery of Brandt-Daroff exercises were needed, including more supervised time with patients and provision of the exercises in an alternative format, such as video. Research supports this approach, suggesting vestibular rehabilitation delivered digitally was superior to usual care (53), whilst a qualitative study in stroke patients noted exercises in video format facilitated rehabilitation (54)

### Training for healthcare professionals and funding for trial activities

Healthcare professionals noted that although the training provided was beneficial, it was front loaded. To maintain interest, competency levels, and ensure rotational staff are trained, future trials might benefit from having more continuous training. Kerber et al. (2020) used a multifaceted educational intervention to train healthcare professionals to manage BPPV in Emergency departments (55). Authors similarly front-loaded training but also included decision aids, a local BPPV champion, and follow-up educational sessions. A comprehensive package as noted above, alongside pre-trial input from therapists, may be beneficial. Finally, therapists noted engaging in research leadership roles during the trial facilitated engagement and development of skills and a research culture within the local team. Indeed, literature notes role extension can provide career opportunities, increased job satisfaction, and motivation (56,57). Furthermore, a recent review on the value of allied health professional research engagement noted improvements to care processes, through facilitation of person-centered changes in knowledge, skills, and attitudes and through organizational centered changes, such as embedded research time within clinician roles (58). This evidence sits alongside the wider national strategy of increasing research capacity and capability among allied health professionals (59,60) and should be considered integral for a future trial.

## Limitations

This study has several limitations. Although an independent researcher interviewed healthcare professionals to reduce bias, patient participants were interviewed by the chief investigator, thus potentially introducing some participant bias. An independent researcher could have also been employed to interview patient participants; however, this may not have resulted in such a high consent rate as patients were opportunistically recruited after their final follow-up appointment with the chief investigator. This study did not gather the views of family members with a relative (or friend) participating in the trial. Future studies could consider this given the need to gain consultee consent for those lacking capacity. BPPV was diagnosed via established criteria (18) which does not mandate the use of videonystagmography. Data on the accuracy and consistency of assessment and treatment procedures are available in the published trial paper (21).

## Conclusions

Patients and healthcare professionals expressed positive views about participating in the study and were optimistic about a future trial. Recommendations were made for modifications to the trial protocol as well as trial delivery. The in-depth data gathered in this qualitative study will inform clinical practice guidelines, as well as shaping a future, larger randomized controlled trial.

## Supplementary Material

Supplemental Material
