# Supplementary material for: What are the views of those participating in a trial investigating acute post-traumatic benign paroxysmal positional vertigo? A qualitative study
Source: Brain Inj. 2024 Dec 3;39(5):400–9. doi: 10.1080/02699052.2024.2435952 (PMC11934952; doi:10.1080/02699052.2024.2435952)
Supplement: Supplemental Material [file IBIJ_A_2435952_SM2472.docx]

**Supplementary material**

**Supplementary Material 1 – Healthcare professional Interview topic guide**

**NHS Healthcare Professional Interview questions**

**Title: A qualitative study exploring the experience of taking part in a feasibility study investigating different treatments for BPPV in acute TBI.**

Chief Investigator: Rebecca Smith, Imperial College, Division of Brain Sciences, Charing Cross Hospital Campus, W6 8RF. Tel: 02033117042

**Introduction**

Thank you for agreeing to participate in this interview. The aim of the interview is to explore your experiences of being involved in the feasibility trial. Your answers will be anonymous and will remain confidential. The interview will be audio recorded and I may take some notes whilst you are talking. Please keep in mind that you do not have to answer any questions you do not feel comfortable with, and we can stop the interview at any time.

**Opener question - ALL**

What was your general experience of taking part in the trial?

**Background questions - ALL**

1. Could you tell me a bit about your role was in the feasibility trial? (i.e., screening, and consenting patients, assessment and treatment, outcome measures)

**Social professional role and identity – THERAPIST ONLY**

1. How did taking part in this study fit into your role as a therapist? (i.e. did you feel as a therapist you were the right person to do this?)

**Beliefs about capabilities – THERAPIST ONLY**

1. How did the assessment and treatment components of the trial fit within your current workload? (i.e. in terms of time? was this acceptable? Do you think this would be feasible to continue with in a longer trial?)
2. How would you rate your ability to diagnose and treat BPPV? (i.e. did you feel you had sufficient training?)

**Skills & Knowledge - Question 5, 8 & 9 THERAPIST ONLY, Question 6 & 7 ALL**

1. How did you find the completing the assessments? (were there any particular patients it was difficult to assess? Do the procedures need to be refined or adapted to make it more acceptable or relevant for future use?)
2. What are your views on the three different treatments we used in the trial? (i.e. did you understand why we used three different treatments? Were you comfortable with this?)
3. What are your views on patients being randomised to one of three different treatments? (what do you think patients felt about being randomised? We have ‘get out’ clauses in the protocol – i.e. patients being able to withdraw and an option for patients to see a Consultant specialising in dizziness if they need to when the study finishes – did these clauses make you feel any differently about randomising patients to the Brandt Daroff or Advice groups?)
4. What was your experience of delivering the treatments as per the protocol? (i.e could you always complete all the treatments and re-assessments? If not why not? did patients decline to complete treatments?
5. If you treated patients in the Brandt Daroff or advice group, were you always able to deliver two treatment sessions? (If not, why not?)
6. Similarly, with patients in the Brandt Daroff or Advice group, were you always able to re-assess patients after treatment? (If not, why not?)
7. What do you think the participants felt about the interventions? (i.e could they understand what you were asking them to do? Could they tolerate the interventions?)

**Beliefs about consequences - THERAPIST ONLY**

1. What were the consequences of the therapy team diagnosing and treating these patients during their hospital stay? (were there any benefits to the patients? To the team?)
2. How do you feel about treating these patients in the acute stage of their injury? (i.e was the acute setting an acceptable time/place to treat BPPV patients or would it be better to wait until they are out of hospital?)

**Environmental context and resources - ALL**

1. Were there clear communication channels if you were unsure of how to do something? (i.e was there adequate support if you needed it?)
2. This study is being run at different sites, have there been any specific barriers or facilitators that made it easy or difficult to participate in the trial? (i.e local or national guidelines about managing dizziness following head injury? would it be useful to have one therapist or nurse whose role just involved completing the non-clinical research activities?)

**Social influences - THERAPIST ONLY**

1. How did your colleagues view you taking on the role of diagnosing and treating BPPV? (other therapists/doctors/nurses/ward managers)

**Emotion - THERAPIST ONLY**

1. Can you describe any situations in which you were worried about managing BPPV in this population? (i.e medically/professionally/emotionally? Was there anything that made you feel uncomfortable?)

**Behavioral regulation - THERAPIST ONLY**

1. Outside of the trial are there any factors that would encourage you to continue with screening for BPPV? (local or national guidelines? Role models?)
2. Is there any further training that you or your team may require? (prompt: any procedures/guidelines/ways of working)

**Trial design questions - ALL**

1. What are your views on the overall design of the trial? (i.e. how patients were selected, the treatments that were used, the number and timing of the follow ups etc.)
2. How did you find recruiting patients onto the trial? (i.e were there any specific barriers to recruiting, if so what were they? Did patients have sufficient cognitive skills to consent? Did we exclude too many patients? Did you have to exclude patients for a particular reason?)
3. What are your views on the outcome measures that the patients were asked to do? (i.e were they relevant? Were you able to complete the outcome measures in a timely manner? Do you think we change remove some? If so which ones?)
4. What are your views on this study being run as a full randomised controlled trial? (i.e could this be run in your setting again? Could it be run in other hospitals for a longer period? Could it run on other wards i.e. with head injury patients in A&E areas?)
5. What are your suggestions on how to improve the study? (Can you give practical examples of what we should do to improve it? i.e. change the paperwork, change REDcap?)

Lastly, is there any further you would like to add which we have not covered?

**Thank you for participating in this interview.**

**Supplementary material 2 – Patient interview topic guide**

**Participant Interview questions**

**Title: A qualitative study exploring the experience of taking part in a feasibility study investigating different treatments for BPPV in acute TBI.**

Chief Investigator: Rebecca Smith, Imperial College, Division of Brain Sciences, Charing Cross Hospital Campus, W6 8RF. Tel: 02033117042

**Introduction**

Thank you for agreeing to participate in this interview. The aim of the study is to explore your experience of participating in the dizziness in TBI feasibility trial. Your answers will be anonymous and will remain confidential. The interview will be audio recorded and I may take some notes whilst you are talking. Please keep in mind that you do not have to answer any questions you do not feel comfortable with, and we can stop the interview at any time.

**Motivation and Goals/Memory and Decision Making**

1. Why did you decide to take part in the trial? (i.e what was your motivation? please discuss any specific factors?)

**Knowledge**

1. How did you find the process of being asked to take part in research after your head injury? (i.e. how were you approached and consented?)
2. What did you think about the way in which we communicated with you? (how well did we explain the study?)
3. How well did you understand the trial from the information you received? (i.e. was there enough, was it easy to understand?)

**Emotion**

1. How did you feel about being randomly allocated to a treatment group? (e.g. Did it worry you? Did you feel it had any impact on your treatment/ recovery?)

**Environmental context and resources**

1. How do / did you feel about your dizziness being diagnosed in the hospital setting? (i.e was this timely? would this have been better done in the community? How did it fit in with the other care you received in hospital?)
2. What changes would you make to how your dizziness was diagnosed and treated which might help patients in a future trial?
3. What are your views on the treatments provided in the trial? (i.e were any sessions uncomfortable/ made your symptoms worse; did you decline to take part in any of the interventions?)
4. How capable did you feel about doing the treatments? (i.e. did the patients believe they could comply with what was being asked of them
5. If there were problems, were communication channels clear and easy to access?

**Skills / Beliefs about capabilities (of the clinicians)**

1. What was your experience of the staff delivering the treatments? (i.e. were the therapists the right staff to deliver the treatments? Were they perceived to be adequately trained?)

**Behavioural regulation**

1. If Brandt-Daroff exercise group – how did you keep up with the exercises prescribed to you by the ward team? (would you have any tips for future patients?)

**Beliefs about consequences**

1. Were there any benefits to being diagnosed and treated in the hospital? (i.e any benefits during the hospital stay and following discharge – were those tangible i.e. falls, going back to daily life, work etc.)
2. Were there any negative consequences of being diagnosed and treated in the hospital?

**Trial design**

1. What was your experience of attending the follow up appointments? (i.e did you miss any? If so, why? Were they conveniently located?)
2. What was your experience of the questionnaires and tests you were asked to do? (i.e were they relevant to you; were they burdensome / too long / difficult to understand or complete?)
3. If we were to run this trial on a larger scale; what sort of things would you tell us to change?
4. Do you think this sort of research could run in other hospitals or other wards?

**Social influences**

1. How did your family/carer/friends view you being part of this research study?

Lastly, is there any further you would like to add which we have not covered?

**Thank you for participating in this interview.**

**Supplementary material 3 – Example of a charted theme**

| **Identifier** | **Characteristics** | **Facilitators to the study** | **Barriers to the study** | **Positives of clinical change** | **Negatives of clinical change** |
| --- | --- | --- | --- | --- | --- |
| **SG1102210333** | Physio, Site B. Female | **Controllable factors:** Previous split research- clinical role giving background understanding how trials run. Split-research- clinical role facilitating fitting research within clinical caseload. Split role provides flexibility to clinicians., without it would be hard to fit into clinical caseload. Split role funded through orthopaedics not therapies, so support must come from specific consultant within team which is critical to facilitate research within clinical role. Research post created research ethos which facilitated participation in study. Sufficiency and flexibility within clinical team to assess patients. patients. CI is a helpful/approachable/available point of contact for queries. Other members of team provide clinical sessions. Online data entry on REDcap reduces data queries and makes things easier. | **Controllable factors:**  Trust trying to promote research to physios, but approach is nursing-led heavy; provides limited research time and requires clinical bank staff. *'all the funding schemes are set up in a very particular way that doesn’t really suit our profession'.* To gain dedicated research time often requires a full clinical academic route, which is a big commitment and may deter physios from research.  **Uncontrollable factors:**  *'*Initial reluctance to change the original practice of treatment with repositioning manoeuvres. General negative feeling in the physiotherapy team of not treating patients as usual. COVID disrupting flow of research. | **Impact on clinicians:** Physiotherapy team enjoyed the ownership of research. Early involvement with study as PI (designing protocol) was rewarding and helpful in improving independent research skills for ongoing research at St Georges. Being given more responsibility within research was a *'learning curve'* but a good, helpful experience. *'raising the profile of research within physios was quite a nice challenge'.* Increasing interest in the study was fulfilling -> *'it was quite nice to get more people on board with that and people were actually getting excited to be part of it so that was nice to see'.* Raising (especially rotational) staff awareness of BPPV*.* | **Impact on clinicians:** *'trial activity takes longer than you think it’s going to'.* Research activities can be time consuming, effecting clinicians day. |
| **SG25022211500** | Physio, Site B, Female | **Controllable factors:**  Sufficiency within clinical team. Approachable PI and CI to answer queries. General engagement in study from neuro trauma coordinator and team facilitated participation in study. Research associate who is also physiotherapist is a facilitator to study. | **Uncontrollable factors:** Reluctance to change routine practice -> *'I have had a few times when I have wanted to treat patients because it’s, by taking part in this study, we are changing what we would normally do at St Georges'.* Study is difficult to explain to patients. | **Impact on clinician:** Learned new skills and knowledge. Knowledge and practice has aided increase in confidence. | **Impact on clinicians:** Change of practice raised challenges within team. Fitting in research activities (consent) could delay treating therapist, slowing patient care down, and caused some patients to be missed*.* |
| **SM0402211211** | Physio, Site A, Female | **Controllable factors:** Research nurses support with research specific activities(time consuming) e.g. consenting, outcome measures facilitate fitting study in with clinical caseload. Training for research activities and practice aided participation and increased efficiency. Written information sheets help with standardised BPPV management. CI is available for queries.  **Uncontrollable factors:** Familiarity with BPPV assessment not increased workload and facilitated participation in study. | **Uncontrollable factors:** '*Nature of trauma it's very variable'*. Caseload varies, so time barriers are present sometimes -> *'there’s times when we have ample time, and we can spend loads of time screening and doing stuff for patients for the trial. But there’s other times, where our caseload has been so high and finding times to do it has been more challenging.'* | **Impact on clinician:** Study has increased patients (no overt BPPV symptoms) being assessed for BPPV, otherwise missed in routine clinical management. Good opportunity to upskill team. Study proformas contain more detail than general notes creating learning opportunity*.* **Impact on patients:** Patients are enthusiastic and gives them something to be involved in. Patients having a diagnosis earlier gives reassurance | **Impact on clinicians:** Fitting in research activities in mild head injury in one day can cause delay in discharge for patients and rush for clinicians. Second therapist is sometimes needed to facilitate treatment positions, taking time out of their clinical day. |
| **SM0402211100** | Occupational therapist, Site A, Female | **Controllable factors:** Research nurses helped save time by consenting patients Initial education surrounding rationale of study and impact on patients is important in motivating teams for study, as there is natural variation in levels of interest amongst team. Continued education throughout trial facilitator to engage with study. Training sessions with neuro-otology team helpful for more ambiguous diagnosis. Accessibility of point of contact for queries is important. Reassurance that there are experts who can be asked about complex cases is a facilitator. | **Uncontrollable factors:**  COVID interruptions caused loss of research nurses, causing therapists to struggle to fit in research activities within clinical day. Belief that for any trained therapist in vestibular with manoeuvres would be difficult to change perspectives of treatments and requires adjustment to have equipoise within study, because there is a lack of evidence base. | **Impact on clinicians:**  Practical and theory knowledge/skills gained. Ability to diagnose and treat BPPV improved. Enjoyment out of process of learning new skill of treatment. Rewarding and motivating as a clinician to improve patient outcomes*.* **Impact on patients:** Increased knowledge surrounding medications improved patient experience as they are given correct medication more promptly. Study could end up speeding up discharge |  |
| **RN2502211130** | Research nurse, Site A, Female | **Controllable factors:** CI point of contact for queries. | **Controllable factors:**  Miscommunications occurred because separate teams working on the same thing. CI could have communicated more clearly feedback to teams e.g. outcome measures. | **Impact on clinicians:**  Study is for a PhD and helping CI. Good experience to do assessments as clinical research nurses. Working within team has been a good experience. Study will also help patients. |  |
| **RN2502211100** | Research nurse, Site A, Female | **Controllable factors:**  *'the CI you know she communicates quite well. She emails us and we emailed her back with queries'* |  |  |  |
| **RN2502211140** | Research nurse, Site A, Female | **Controllable factors:**  Clear communication channels if unsure on how to do something. CI provided training and education on BPPC and rationale behind study **Uncontrollable factors:**  Therapy team is friendly and supportive. | **Uncontrollable factors:**  COVID caused miscommunications e.g. outcome measure. COVID effected research nurses-> | **Impact on clinicians:** *'It’s been a good networking opportunity. We have got to know the therapists on the ward really well. So that’s been a really, really positive thing.'* |  |
| **RN260211130** | Research nurse, Site A, Male | **Controllable factors:**  Lead research nurse and CI was point of contact for queries. Physiotherapists were also helpful for answering queries. | **Controllable factors:**  Physiotherapists telling research nurses of patients late in day causing disruption to organisation of day. Miscommunication between research nurses and therapists. |  | Takes two research nurses to complete outcome measures, so if they are told late by the therapists, it disrupts the day. |
| **KC2809210900** | Occupational therapist, Site C, Male | **Controllable factors:**  CI was very supportive. Therapy team received face-to-face updated training to e ensure knowledge was standardised. Every aspect of study was delivered by therapy teams for example consenting, outcome measures, assessment, and treatment. Clear communication channels. | Varying level of experience amongst the clinical team.  **Uncontrollable factors:** Some examples include needing approval from a local committee, also COVID impacting pausing the study at various sites. Felt time put in setting up study was unacceptable, but this is mostly due to governance | **Impact on clinicians:** Benefited from study training to refresh skills. Perspective that therapy team saw the study as an opportunity*. 'We actually captured survey data pre- and post-completion of the training which showed a huge increase in confidence around assessment and treatment.'* |  |
| **SG1910211100** | Physio, Site B, Female | CI and PI provided support, and both were happy to help. Neuro physios signed off on delegation lob who clinician could ask for supervision with neuro patients. Team already had an awareness and understanding of BPPV which aided in carrying out the study. | Staffing was an issue to complete second treatments and physical outcome measures. Extra research role but is split between 10 studies, so is not enough time. | Helped to understand the condition better, thus improving communication to patient about BPPV. It gives people the opportunity to gain more experience in treating patients. Gives team more understanding of advice and exercises | Only worried about managing BPPV is patient had severe BPPV and was sent home. |
| **KC1808211200** | Occupational therapist, Site C, Female | Team of staff who were available to help with trial processes. Trial processes did not take much time out of clinical day as it was very straightforward. CI was able to answer questions. Three training opportunities to upskill. | Difficult to maintain competency if there is not enough patients to practice on. Main barrier is clinical capacity, some days are busier than others. | Trial enabled clinician to get experience in treating and assessing dizziness, which they increased confidence. Being able to assess and treat BPPV helps with discharge planning and the support needed at home. |  |
| **SM2009211500** | Physio, Site A, Female | Research team doing consent alleviated pressure. | Time pressures to complete paperwork and complete the process (now knowing patient has a diagnosis/problem). Time to chase research team to do outcome measures. Research nurses prioritised other studies so were delayed in completing their procedures. Meant juggling/waiting/inefficiencies. Consent late in the day or late on in admission problematic. | Was able to see patients where treatments had improvements so highlights that team were missing gap in care. Now part of routine practice to proactively look for BPPV. Now prioritise it. Can do something short term that has an impact on the patient longer term. Patients have reported symptomatic improvement with active treatment. More awareness of how to assess and treat these patients. | Provocation of symptoms and not always curing it. Repeated manoeuvres feels mean. If natural resolution feels mean to provoke symptoms when not necessary |
| **KC1808211000** | Occupational therapist, Site C, Female | Had quite a lot of support from managers to prioritise BPPV study. Thinks level of training was fine. Could contact site PI or CI for help if needed. Interest and excitement from team members. | Barriers might be confidence with assessment and treatment for OTs - technical language can sound quite intimidating. Sometimes was difficult to know how to prioritise research tasks and clinical tasks. Some political dynamics between physios/OTs/managers | Been an eye opening experience. |  |
| **SM2506211211** | Physio, Site A, Male | Felt had sufficient training. Spent quite a lot of time with the CI. Funding for RN's to complete consent alleviated pressure on therapists. The rest of the tasks were easy to manage within the team - helpful having a few people trained up to split workload. More training and experience and better understanding of the research helped with feeling ok with random allocation. | Time to complete paperwork and upload it to the system. Paper based things aren't helpful. Better to do it online/computer based. Patients based on outlying wards and having relevant staff to assess/treat was more difficult. More that are trained, the better. | Thinks assessing earlier and have increased awareness amongst medical team that therapists can treat. Medical team less likely to send patients home and discuss with therapists now instead. Increased awareness amongst therapists to assess vestibular function. |  |
| **KC1908211100** | Physio, Site C, Female | Interest in vestibular so quite exciting to be a part of it. Helpful for patients - interested in what’s going on / their symptoms. Assessment already doing so fits in well with what was already doing. Assessment and treatment quick and easy to do. patients happy to take part | Sometimes not enough time to do everything in one day - sometimes challenging if going home that day. Having to wait for outcome measures to do treatment can be tricky instead of doing treatment straightaway. Sometimes busy days when not able to get to patient to do assessment. Not having enough staff trained - annual leave occasionally can mean an issue. | may save money as picking things up earlier. Good learning experience for therapists - feels like doesn't do it enough in acute setting. |  |
